# The impact of psychophysiological well being on executive functions among anaesthesia residents

**DOI:** 10.1097/EJA.0000000000002106

**Published:** 2024-12-09

**Authors:** Annalisa Boscolo, Luca Queirolo, Paolo Navalesi, the PADOVA Stress Group

**Affiliations:** From the Department of Medicine - DIMED, Section of Anaesthesiology and Intensive Care, University of Padova (BA, PN), Institute of Anaesthesia and Intensive Care, Padova University Hospital (BA, PN), Thoracic Surgery and Lung Transplant Unit, Department of Cardiac, Thoracic and Vascular Sciences (BA), Section of Clinical Dentistry, Department of Neurosciences (QL) and Department of Philosophy, Sociology, Education and Applied Psychology, University of Padova, Padova, Italy (QL)

Editor,

Because anaesthesia practice requires managing stress and anxiety while upholding critical cognitive functions,^1^ this specific workplace represents a unique scenario to investigate the relationship between executive functions and work-related stress and anxiety. In particular, anaesthesia practice may be psychologically challenging for residents who first approach such a demanding environment. Indeed, stress and anxiety are quite common in anaesthesia residents due to several factors, such as the responsibility for the safety of patients and the occurrence of unexpected emergencies.^2^ Accordingly, between 1 January and 31 March 2024, we enrolled residents, attending from the second to the fourth year of residency in Anaesthesia and Intensive Care Medicine at the University of Padua, aiming to investigate whether and how executive functions change after work shift; and how different levels of physiological arousal, anxiety, stress and worry may influence cognitive performances. Specifically, each participant was followed from the beginning to the end of a diurnal shift. Exclusion criteria were known cardiovascular or psychiatric disorders.

The following tests were applied:

(1) the ‘revised’ Tower of London Test (TOL-R), evaluating the cognitive performance both preshift and postshift;^3–5^
(2) electrodermal activity (EDA) and skin conductance response (SCR/min), investigating the variation of the electrical conduction in response to stress-induced sweat secretions, providing reliable evaluation of physiological arousal during work;^6,7^
(3) the Visual Analog Scale (VAS) for anxiety, recorded at baseline and at the end of each shift, and the State-Trait Anxiety Inventory (STAI-Y-1 and 2) tests, administered only at baseline;^8^
(4) the Perceived Stress Scale (PSS)-10 before work shift, assessing the perceived stress level in the last month;^9^
(5) the Penn State Worry Questionnaire (PSWQ) before work shift, measuring pathological worry.^10^

This prospective study, was approved by the Ethical Committee of the Department of General Psychology, University of Padua (reference n.3274/2019 on 05 November 2019, Chairwoman Professor A. Falco) and registered on ClinicalTrial.gov (NCT06200792), and written in accordance with the ‘Strengthening the reporting of observational studies in epidemiology’ reporting checklist (SDC 1).

Continuous data are presented as mean ± standard deviation, when normally distributed, or as median [interquartile range, IQR], when non-normally distributed. Categorical data are reported as numbers and percentages. Each participant was followed from the beginning (prework shift or ‘baseline’) to the end of a day shift (post-work shift). For investigating potential changes in TOL-R scores after work shifts, and considering the data as paired, we needed a sample size of 34 participants to reliably detect, with a probability greater than 0.8, an effect size of delta at least 0.5, assuming a two-sided criterion for detection that allows for a maximum type I error rate of alpha = 0.05. For comparing paired data, linear mixed-effects models were used to evaluate the association between each outcome and time, adjusted for age and year of residency. The model included the individual resident as a random effect and the effect of time as a fixed factor. The estimates, standard errors (SEs) and *P* values of the associations were reported. For comparing unpaired data, unpaired *t*-tests or Mann–Whitney tests were performed. Analyses are performed using R Software 3.5.2 and GraphPad Prism 8.0. All tests are two-tailed, and statistical significance is defined by *P* value less than 0.05.

Among 58 residents attending our Institution, 42 (72.4%) participants (20 men, 22 women; mean age was 31 ± 5 years) met study criteria; one male participant was excluded because of hypertension. Eleven (27%) participants attended the second year, 15 (37%) the third year and 15 (37%) the fourth year of residency.

TOL-R, EDA, SCR/min and psychological evaluations are reported in Tables 1 and 2, respectively. Compared with baseline, TOL-R score improved after work shift (adjusted estimate 3.20; SE 0.73; *P* value <0.001), whereas EDA (adjusted estimate -0.80; SE 0.44; *P*-value 0.074), SCR min^−1^ (adjusted estimate −0.38; SE 0.76; *P* value 0.617) and self-reported VAS (adjusted estimate −0.54; SE 0.34; *P* value 0.121) were similar (Table 1).

**Table 1:** Prework and postwork shift measurements

	Preshift	Postshift
TOL-R	17 ± 5	21 ± 5
	adjusted estimate 3.20; SE 0.73; *P* < 0.001	
EDA, μS	4.32 [2.90–6.96]	4.18 [2.63–5.83]
	adjusted estimate −0.80; SE 0.44; *P* = 0.074	
SCR min^−1^	7.50 [5.66–9.00]	6.66 [4.33–8.58]
	adjusted estimate −0.38; SE 0.76; p 0.617	
VAS	adjusted estimate −0.54; SE 0.34; p 0.121	

Preshift															
	VAS			STAI-Y-1			STAI-Y-2			PSS-10			PSWQ		
TOL-R	≥5	<5	*P*	≥40	<40	*P*	≥44	<44	*P*	≥14	<14	*P*	≥40	<40	*P*
	21 ± 2	16 ± 5	0.005	16 ± 6	19 ± 5	0.148	16 ± 5	18 ± 5	0.201	18 ± 5	17 ± 5	0.508	17 ± 6	18 ± 5	0.856

Data are shown as mean difference and SD or as median and [interquartile range]. TOL-R, ‘revised’ tower of London; VAS, visual analogue scale; SD, standard deviation; STAI-Y, state-trait anxiety inventory; PSS-10, perceived stress scale; PSWQ, Penn State Worry Questionnaire; *P*, *P* value; EDA, electrodermal activity; SCR min^−1^, skin conductance response; VAS, visual analogue scale; SE, standard error.

**Table 2:** Psychological measurements

			Abnormal results	
Condition	Test	Overall	Cut-off	*N* (%)
Anxiety	VAS preshift	4.51 ± 2.13	≥5	20 (48.8%)
	VAS postshift	3.56 ± 2.19	≥5	13 (31.7%)
	STAI-Y-1	39 ± 9	≥40	18 (43.9%)
	STAI-Y-2	40 ± 9	≥44	11 (26.8%)
Stress	PSS-10	16 ± 6	≥14	26 (63.4%)
Worry	PSWQ	42 ± 13	≥40	19 (46.3%)

Data are shown as mean ± standard deviation, or as number (*N*) and percentage (%). VAS, visual analogue scale; STAI-Y, state-trait anxiety inventory; PSS-10, perceived stress scale; PSWQ, Penn State Worry Questionnaire.

In respect to participants with lower self-reported anxiety, residents with pre-shift VAS score greater than 5 recorded better baseline TOL-Rs (21 ± 2 vs. 16 ± 5, *P* value 0.005), whereas no differences were registered comparing residents with normal *vs*. abnormal results in all other psychological evaluations (Table 1).

Finally, 14 residents (34%) experienced intraoperative emergencies, defined as an unexpected exposure to anaesthetic emergencies or to emergent surgical procedures during the shift, and reported worse cognitive performances and greater anxiety, as shown in SDC 2.

To the best of our knowledge, this is the first study showing the increase in cognitive performance after diurnal work shift among anaesthesia residents. This finding may be justified by the Yerkens and Dodson's Law, which explains that a moderate level of activation and anxiety leads to better performance, while, on the opposite side, an exaggerated activation, mediated by excessive anxiety, worsens the performance.^6,10,11^ Interestingly, the lower increase of TOL-R in participants exposed to intraoperative emergencies may suggest the importance of a proper learning curve. Indeed, we hypothesise that residents do not have the same trained skills as expert consultants, therefore, especially in case of unexpected scenarios, such as intraoperative emergencies, this ‘work immaturity’ may negatively affect cognitive function and anxiety. Notably, as in our study, TOL-R performances were affected by the occurrence of intraoperative emergencies and higher self-reported anxiety levels, we speculate that environmental-related conditions and current psychological characteristics (i.e. anxiety perception preshift and postshift) may be more important than participants’ remote psychological history (assessed by conventional psychological tests) in influencing cognitive performance.

The present study has some limitations that must be mentioned. Firstly, it is a single-centre pilot study, only focused on anaesthesia residents, bearing all the limitations of this research design. Secondly, the sample size is limited, with potential issues of reproducibility, considering other residency programs.^5^ Thirdly, we investigated only diurnal work shifts with the aim to limit the influence of factors such as severe sleep deprivation, challenging night shifts or prolonged in-hospital calls on our results, as largely described in literature.^12–14^ Lastly, we cannot exclude a potential role of different factors, not explored by our tests, on cognitive performance in this specific workplace.

In conclusion, in a cohort of moderately anxious, stressed and worried anaesthesia residents, cognitive performance improved after work shift but to a lesser extent in case of intraoperative emergencies. Moreover, a higher anxiety perception and a moderately increased state of physiological arousal positively affected cognitive function.

## Supplementary Material

Supplemental Digital Content

## Supplementary Material

Supplemental Digital Content

## Supplementary Material

Supplemental Digital Content
