# Supplementary material for: The impact of psychophysiological well being on executive functions among anaesthesia residents
Source: Eur J Anaesthesiol. 2024 Dec 9;42(4):366–8. doi: 10.1097/EJA.0000000000002106 (PMC11872262; doi:10.1097/EJA.0000000000002106)
Supplement: Supplemental Digital Content [file ejanet-42-366-s002.docx]

# **SUPPLEMENTAL DIGITAL CONTENT**

**SDC 2. Additional data about intraoperative emergencies.**

Residents who experienced unexpected anaesthetic emergencies or were exposed to emergent surgical procedures during the shift (14, 34%) recorded a lower increase in TOL-R scores after work (TOL-R delta (%)* 8 ± 13), as compared to those residents not exposed to emergencies (TOL-R delta (%) 42 ± 7, p-value **0.016**); while no differences were found considering EDA (EDA delta (%) 20 (IQR 17 to 35) vs 2.24 (IQR -37 to 30), p-value 0.153) and SCR/min (SCR/min delta (%) 0 (IQR -23 to 32) vs -27 (IQR -47 to 60) (p-value 0.456). With regards to VAS scores, residents who experienced anaesthetic emergencies or were exposed to an emergent surgical procedure during the shift reported greater VAS values after the work shift (VAS delta (%) 19 ± 6), as compared to residents not exposed to emergencies (VAS delta (%) -20 ± 4, p-value **0.023**).

*: To clarify, ‘delta’ was defined as the percentage difference between post- and pre-shift measurements, considering pre-shift values as reference (i.e., 100%).
