# Supplementary material for: The impact of psychophysiological well being on executive functions among anaesthesia residents
Source: Eur J Anaesthesiol. 2024 Dec 9;42(4):366–8. doi: 10.1097/EJA.0000000000002106 (PMC11872262; doi:10.1097/EJA.0000000000002106)
Supplement: Supplemental Digital Content [file ejanet-42-366-s003.docx]

**APPENDIX 1**

Members of the **PADOVA Stress Group**, to be searchable through their individual PubMed records, are listed below:

1. Section of Clinical Dentistry, Department of Neurosciences, University of Padova - Padova, Italy (Zanette Gastone, MD, Prof, Stellini Edoardo, MD, Prof)
2. Department of Medicine - DIMED, Section of Anaesthesiology and Intensive Care, University of Padova - Padova, Italy (Medici Francesca, MD, Bassi Mara, MD, Fincati Valentina, MD, Biscaro Martina, MD, Della Paolera Michele, MD, Monteleone Francesco, MD, Pandolfo Giovanna, MD, Congedi Sabrina, MD, Pistollato Elisa, MD, Mormando Giulia, MD, Perona Matteo, MD, Pettenon Giovanni, MD, Giacon Tommaso Antonio, MD, Zanon Paola, MD, Toma Francesca Maria, MD, Coniglio Giordana, MD)
3. Institute of Anaesthesia and Intensive Care - Padova University Hospital - Padova, Italy (Sella Nicolò, MD, Pettenuzzo Tommaso, MD, Zarantonello Francesco, MD, De Cassai Alessandro, MD)
4. School of Medicine, University of Padua, Padova, Italy (Magrini Andrea)

**COMPETING INTERESTS:** The authors belonging to the **PADUA Stress Group** have no competing interests to declare.

**CONTRIBUTIONS:**  ZG, MF, FV, BM, DCA, PT, ZF, DPM, MF, PG, CS, PS, MG, PM, PG, GTA, ZP, TF, GC, SE and MA substantially contributed to the study design, data interpretation and the writing of the manuscript. ZG, MF, FV, BM, DCA, PT, ZF, DPM, MF, PG, CS, PS, MG, PM, PG, GTA, ZP, TF, GC, SE and MA contributed to data collection, interpretation, and manuscript writing. All members of Padova Stress Group conceived, performed and guaranteed the accuracy of data analysis.
